# Supplementary material for: Dengue transmission heterogeneity across Indonesia’s archipelago: Climate-driven spatiotemporal patterns and policy implications
Source: PLoS Negl Trop Dis. 2026 Mar 17;20(3):e0014135. doi: 10.1371/journal.pntd.0014135 (PMC13004528; doi:10.1371/journal.pntd.0014135)
Supplement: S1 Appendix — Table A: Province names and their corresponding numerical indices fro reference to all maps in the main figures. Table B: Annual national dengue incidence by epidemic year (July-June), with z-scores relative to the study period mean. Epidemic year “2015-16” represents July 2015 to June 2016. Major outbreak years are defined as z-score > 1 (more than one standard deviation above the mean). Table C: Silhouette statistics for different numbers of clusters (k = 2 to k = 15) for all 34 provinces. The selected number of clusters (k = 12) accounts for both silhouette statistics and balance in cluster sizes. Table D: Silhouette statistics for different numbers of clusters (k = 2 to k = 15) for western provinces including Kalimantan (22 provinces). The selected number of clusters is k = 10. Table E: Silhouette statistics for different numbers of clusters (k = 2 to k = 15) for western provinces only (Sumatra and Java-Bali, 17 provinces). The selected number of clusters is k = 4, representing the cleanest cluster structure. Table F: Mean DTW distance from each province to other provinces in the same geographic region, with z-scores calculated within each region. Provinces with |z| > 1.65 are flagged as temporal outliers whose dynamics deviate substantially from their regional pattern. Table G: Spearman correlations between large-scale climate indices (ONI and DMI) and dengue incidence by geographic region, using epidemic year aggregation (July-June). Table H: Sensitivity analysis (COVID-19): comparison of province-level dengue phase lags between the full study period (2010–2024) and the pre-pandemic period (2010–2019). Table I: Sensitivity analysis (COVID-19): comparison of climate-dengue phase coherence and qualifying status (coherence ≥0.85, phase lag ≥ 0) between the full study period (2010–2024) and the pre-pandemic period (2010–2019), for precipitation and temperature. Table J: Sensitivity analysis (COVID-19): comparison of DLNM-derived cumulative relative risks at wavele [file pntd.0014135.s001.pdf]

S1 Appendix: Dengue transmission heterogeneity across Indonesia's archipelago: climate-driven spatiotemporal patterns and policy implications

Bimandra Djaafara, Iqbal Elyazar, Fadjar SM Silalahi, Asik Surya, Agus Handito, Burhannudin Thohir, Desfalina Aryani, Mushtofa Kamal, Aditya L Ramadona, Dyana Gunawan, Hipokrates, Anzala Khoirun Nisa, Edi Prianto, Iriani Samad, Agus Sugiarto, Nuno R Faria, Kimberly Fornace, Hannah E Clapham, Swapnil Mishra

2026-03-12

Table of Contents

1 Supplementary Tables **3**
- 1.1 Table A: Province Reference Index . . . 3
- 1.2 Table B: Annual Dengue Incidence by Epidemic Year . . . 4
- 1.3 Table C: Silhouette Statistics for Cluster Optimization (All Provinces) . . . 5
- 1.4 Table D: Silhouette Statistics for Cluster Optimization (Western Provinces + Kalimantan) . . . 6
- 1.5 Table E: Silhouette Statistics for Cluster Optimization (Western Provinces Only) . . . 7
- 1.6 Table F: Province Dissimilarity Within Regions . . . 8
- 1.7 Table G: Regional Correlations Between Climate Indices and Dengue Incidence . . . 9

2 COVID-19 Pandemic Sensitivity Analysis **10**
- 2.1 Methods . . . 10
- 2.2 Results . . . 10
  - 2.2.1 Table H: Phase Lag Comparison . . . 10
  - 2.2.2 Table I: Phase Coherence Comparison . . . 12
  - 2.2.3 Table J: DLNM Cumulative Relative Risk Comparison . . . 14
- 2.3 Interpretation . . . 16

3 Phase Coherence Threshold Sensitivity Analysis **17**
- 3.1 Methods . . . 17
- 3.2 Results . . . 18
  - 3.2.1 Table K: Threshold Sensitivity Analysis . . . 18
- 3.3 Interpretation . . . 18

4 Supplementary Figures **19**
- 4.1 Figure A: Geographical Regions of Indonesia . . . 19
- 4.2 Figure B: Province Phase Lags with Confidence Intervals . . . 20
- 4.3 Figure C: Comparison of Wavelet-Based and DLNM-Based Lag Estimates . . . 21

4.4 Figure D: Year-by-Year Peak Month Variability (All Provinces) 23
4.5 Figure E: Year-by-Year Peak Month Variability (Western Provinces) 24

Table 1

Table A. Province Reference Index

Index	Province Name	Admin ID
1	Aceh	11
2	North Sumatra	12
3	West Sumatra	13
4	Riau	14
5	Jambi	15
6	South Sumatra	16
7	Bengkulu	17
8	Lampung	18
9	Bangka-Belitung Islands	19
10	Riau Islands	21
11	Jakarta	31
12	West Java	32
13	Central Java	33
14	Yogyakarta	34
15	East Java	35
16	Banten	36
17	Bali	51
18	West Nusa Tenggara	52
19	East Nusa Tenggara	53
20	West Kalimantan	61
21	Central Kalimantan	62
22	South Kalimantan	63
23	East Kalimantan	64
24	North Kalimantan	65
25	North Sulawesi	71
26	Central Sulawesi	72
27	South Sulawesi	73
28	Southeast Sulawesi	74
29	Gorontalo	75
30	West Sulawesi	76
31	Maluku	81
32	North Maluku	82
33	West Papua	91
34	Papua	94

1 Supplementary Tables

1.1 Table A: Province Reference Index

Province names and their corresponding numerical indices for reference to all maps in the main figures.

Table 2

Table B. Annual Dengue Incidence by Epidemic Year

Epidemic Year[1]	Total Cases	Incidence per 100,000	Z-score	Year Type
2010-11	85668	35.68	-0.53	Below average
2011-12	84716	34.83	-0.58	Below average
2012-13	110821	44.97	0.01	Average
2013-14	91108	36.50	-0.48	Average
2014-15	138262	54.70	0.59	Above average
2015-16	185198	72.36	1.63	Major outbreak
2016-17	101528	39.18	-0.33	Average
2017-18	55794	21.27	-1.38	Below average
2018-19	138751	52.27	0.44	Average
2019-20	119912	44.64	-0.01	Average
2020-21	54386	20.01	-1.46	Below average
2021-22	118748	43.19	-0.09	Average
2022-23	124250	44.67	0.00	Average
2023-24	230635	81.99	2.19	Major outbreak

[1]Epidemic year follows July-June pattern to capture continuous outbreak seasons

1.2 Table B: Annual Dengue Incidence by Epidemic Year

Incidence by July-June annual period and their respective z-scores. Epidemic year "2015-16" represents July 2015 to June 2016. Major outbreak years are defined as z-score > 1 (more than one standard deviation above the mean).

Table 3

Table C. Silhouette Statistics (All Provinces)

Number of Clusters	Silhouette Score
2	0.137
3	0.101
4	0.077
5	0.066
7	0.060
12	0.056
8	0.054
13	0.053
9	0.050
14	0.050
10	0.049
15	0.048
6	0.046
11	0.041

1.3 Table C: Silhouette Statistics for Cluster Optimization (All Provinces)

Silhouette statistics for different numbers of clusters (k=2 to k=15) for all 34 provinces. The selected number of clusters (k=12, highlighted) accounts for both silhouette statistics and balance in cluster sizes.

Table 4

Table D. Silhouette Statistics (Western Provinces + Kalimantan)

Number of Clusters	Silhouette Score
2	0.125
3	0.098
10	0.081
9	0.081
11	0.078
8	0.074
13	0.072
12	0.071
14	0.067
6	0.066
4	0.066
5	0.062
7	0.060
15	0.060

1.4 Table D: Silhouette Statistics for Cluster Optimization (Western Provinces + Kalimantan)

Silhouette statistics for western provinces including Kalimantan (22 provinces). The selected number of clusters is k=10 (highlighted).

Table 5

Table E. Silhouette Statistics (Western Provinces Only)

Number of Clusters	Silhouette Score
4	0.127
3	0.114
5	0.103
2	0.102
8	0.087
7	0.086
6	0.084
9	0.077
10	0.069
11	0.057
12	0.047
13	0.039
14	0.032
15	0.023

1.5 Table E: Silhouette Statistics for Cluster Optimization (Western Provinces Only)

Silhouette statistics for western provinces only (Sumatra and Java-Bali, 17 provinces). The selected number of clusters is k=4 (highlighted), representing the cleanest cluster structure.

Table 6

Table F. Province Dissimilarity Within Regions

Region	Province	Mean DTW	Z-score[1]	Outlier
Java & Bali	JAWA TIMUR	86.77	1.76	Yes
Java & Bali	DKI JAKARTA	85.14	0.92	No
Java & Bali	DAERAH ISTIMEWA YOGYAKARTA	83.29	-0.03	No
Java & Bali	JAWA TENGAH	82.70	-0.32	No
Java & Bali	BANTEN	82.22	-0.57	No
Java & Bali	JAWA BARAT	82.03	-0.67	No
Java & Bali	BALI	81.21	-1.09	No
Kalimantan	KALIMANTAN UTARA	91.53	1.53	No
Kalimantan	KALIMANTAN SELATAN	85.18	0.27	No
Kalimantan	KALIMANTAN TIMUR	82.59	-0.25	No
Kalimantan	KALIMANTAN BARAT	81.99	-0.37	No
Kalimantan	KALIMANTAN TENGAH	77.91	-1.18	No
Sulawesi	SULAWESI BARAT	114.06	1.85	Yes
Sulawesi	GORONTALO	106.40	0.24	No
Sulawesi	SULAWESI TENGAH	105.08	-0.03	No
Sulawesi	SULAWESI UTARA	103.05	-0.46	No
Sulawesi	SULAWESI TENGGARA	101.42	-0.80	No
Sulawesi	SULAWESI SELATAN	101.37	-0.81	No
Sumatra	KEPULAUAN RIAU	106.61	1.98	Yes
Sumatra	LAMPUNG	102.18	0.89	No
Sumatra	SUMATRA BARAT	101.31	0.67	No
Sumatra	SUMATRA UTARA	100.05	0.36	No
Sumatra	ACEH	98.82	0.05	No
Sumatra	BENGKULU	97.15	-0.36	No
Sumatra	KEPULAUAN BANGKA BELITUNG	96.70	-0.47	No
Sumatra	JAMBI	94.90	-0.92	No
Sumatra	RIAU	94.79	-0.94	No
Sumatra	SUMATRA SELATAN	93.48	-1.27	No

[1]Z-score calculated within each region. Provinces with $|z| > 2$ flagged as outliers.

1.6 Table F: Province Dissimilarity Within Regions

Mean DTW distance from each province to other provinces in the same region, with z-scores calculated within each region. Provinces with $|z| > 1.65$ are flagged as temporal outliers whose dynamics deviate substantially from their regional pattern.

Table 7

Table G. Regional Climate Index Correlations

Region	N Years	ONI Correlation	DMI Correlation	Better Index
JAVA & BALI	14	0.622	0.024	ONI
KALIMANTAN	14	0.903	0.508	ONI
MALUKU	14	0.288	0.600	DMI
NUSA TENGGARA	14	0.319	0.459	DMI
PAPUA	14	0.785	0.459	ONI
SULAWESI	14	0.833	0.490	ONI
SUMATRA	14	0.675	0.512	ONI

1.7 Table G: Regional Correlations Between Climate Indices and Dengue Incidence

Spearman correlations between large-scale climate indices (ONI and DMI) and dengue incidence by geographic region, using epidemic year aggregation (July-June).

2 COVID-19 Pandemic Sensitivity Analysis

The COVID-19 pandemic (2020-2024) may disrupt dengue surveillance systems across Indonesia. To assess whether our findings are robust to potential surveillance artifacts during this period, we conducted a sensitivity analysis comparing results from the full study period (2010-2024) with a pre-pandemic subset (2010-2019).

2.1 Methods

We re-ran three key analyses using only pre-pandemic data:

1. **Wavelet phase analysis** to extract province-level dengue timing patterns
2. **Climate-dengue phase coherence** to identify provinces where climate reliably leads dengue
3. **DLNM analysis** for qualifying provinces (coherence 0.85, positive phase lag) to estimate cumulative relative risk

We then compared results across the two periods using three metrics: (1) agreement in phase lag estimates, (2) agreement in phase coherence, and (3) agreement in effect direction (elevated vs. protective) for DLNM-derived cumulative relative risks.

2.2 Results

2.2.1 Table H: Phase Lag Comparison

Comparison of dengue phase lags (timing relative to other provinces) between full dataset (2010-2024) and pre-pandemic period (2010-2019).

Table 8

Table H. Phase Lag Sensitivity to COVID-19 Period[1]

Prov.	Reg.	Lag (F)	LCI (F)	UCI (F)	Lag (PP)	LCI (PP)	UCI (PP)	Diff	Agree
ACEH	Sumatra	-2.23	-3.50	1.12	-2.18	-4.23	0.88	-0.04	Same direction
BENGKULU	Sumatra	0.56	-1.17	1.79	0.08	-1.23	1.74	0.47	Same direction
JAMBI	Sumatra	-1.46	-3.03	2.53	-1.59	-3.47	1.69	0.13	Same direction
KEPULAUAN BANGKA BELITUNG	Sumatra	-1.24	-2.93	2.63	-0.42	-2.39	2.85	-0.81	Same direction
KEPULAUAN RIAU	Sumatra	-2.41	-4.40	-0.41	-2.78	-4.40	0.11	0.37	Same direction
LAMPUNG	Sumatra	-0.25	-1.67	3.12	0.07	-1.90	3.27	-0.32	Different direction
RIAU	Sumatra	-1.93	-3.82	1.67	-2.18	-4.22	0.89	0.25	Same direction
SUMATRA BARAT	Sumatra	-1.03	-2.26	1.62	-1.26	-3.17	2.02	0.22	Same direction
SUMATRA SELATAN	Sumatra	-0.70	-2.16	3.40	-0.28	-2.18	3.07	-0.42	Same direction
SUMATRA UTARA	Sumatra	-2.96	-4.15	-0.58	-3.45	-4.68	-0.68	0.49	Same direction
BALI	Java & Bali	1.30	-0.06	3.54	1.76	-0.19	4.22	-0.46	Same direction
BANTEN	Java & Bali	0.79	-0.62	3.72	1.47	-0.43	3.96	-0.68	Same direction
DAERAH ISTIMEWA YOGYAKARTA	Java & Bali	0.57	-0.84	3.58	1.14	-0.76	4.16	-0.57	Same direction
DKI JAKARTA	Java & Bali	1.40	0.12	3.51	1.80	-0.22	4.33	-0.40	Same direction
JAWA BARAT	Java & Bali	1.33	-0.13	3.56	1.74	-0.28	4.23	-0.41	Same direction
JAWA TENGAH	Java & Bali	0.53	-0.88	3.70	0.96	-1.00	4.04	-0.43	Same direction
JAWA TIMUR	Java & Bali	0.29	-1.28	3.62	0.54	-1.43	3.74	-0.25	Same direction
KALIMANTAN BARAT	Kalimantan	-2.09	-3.45	1.31	-1.85	-3.68	1.43	-0.24	Same direction
KALIMANTAN SELATAN	Kalimantan	-0.91	-2.48	3.08	-0.62	-2.58	2.66	-0.30	Same direction
KALIMANTAN TENGAH	Kalimantan	-0.92	-2.49	3.07	-0.53	-2.50	2.75	-0.39	Same direction
KALIMANTAN TIMUR	Kalimantan	0.26	-1.30	4.17	0.80	-1.17	3.92	-0.53	Same direction
KALIMANTAN UTARA	Kalimantan	0.86	-0.53	2.12	1.69	-0.19	3.97	-0.82	Same direction
NUSA TENGGARA BARAT	Nusa Tenggara	0.36	-1.05	4.01	0.90	-1.00	4.09	-0.54	Same direction
NUSA TENGGARA TIMUR	Nusa Tenggara	-0.53	-1.99	3.57	-0.07	-1.97	3.28	-0.47	Same direction
GORONTALO	Sulawesi	0.21	-1.36	3.25	0.25	-1.71	3.45	-0.05	Same direction
SULAWESI BARAT	Sulawesi	-0.37	-1.25	1.75	-0.64	-2.52	2.64	0.26	Same direction
SULAWESI SELATAN	Sulawesi	0.39	-0.98	3.51	0.70	-1.20	3.96	-0.30	Same direction
SULAWESI TENGAH	Sulawesi	-0.05	-1.41	2.11	-0.33	-2.23	2.62	0.27	Same direction
SULAWESI TENGGARA	Sulawesi	0.34	-1.07	3.88	0.69	-1.21	3.87	-0.35	Same direction
SULAWESI UTARA	Sulawesi	0.21	-1.20	3.64	0.59	-1.31	3.93	-0.38	Same direction
MALUKU	Maluku	-0.32	-1.84	2.21	-0.47	-2.37	2.87	0.15	Same direction
MALUKU UTARA	Maluku	1.03	-0.25	2.37	1.43	0.20	3.35	-0.40	Same direction
PAPUA	Papua	-0.21	-1.57	2.39	-0.33	-2.23	2.94	0.12	Same direction
PAPUA BARAT	Papua	-0.09	-1.47	1.36	-0.07	-1.46	1.25	-0.01	Same direction

[1] F = Full period (2010-2024); PP = Pre-pandemic (2010-2019); LCI/UCI = Lower/Upper confidence interval

2.2.2 Table I: Phase Coherence Comparison

Comparison of climate-dengue phase coherence between full dataset and pre-pandemic period for precipitation and temperature. "Qualified" indicates provinces meeting threshold criteria (coherence 0.85, phase lag 0) for inclusion in DLNM analysis.

Table 9

Table I. Phase Coherence Sensitivity to COVID-19 Period[1]

Prov.	Reg.	Clim.	Lag (F)	Coh (F)	Lag (PP)	Coh (PP)	ΔCoh	ΔLag	Qual (F)	Qual (PP)	Agree
BALI	Java & Bali	Precipitation	2.52	0.96	2.47	0.95	0.00	0.05	TRUE	TRUE	Same
BANTEN	Java & Bali	Precipitation	1.80	0.92	2.02	0.97	-0.05	-0.22	TRUE	TRUE	Same
DAERAH ISTIMEWA YOGYAKARTA	Java & Bali	Precipitation	1.75	0.92	1.85	0.95	-0.04	-0.09	TRUE	TRUE	Same
DKI JAKARTA	Java & Bali	Precipitation	2.54	0.93	2.34	0.94	-0.01	0.19	TRUE	TRUE	Same
JAWA BARAT	Java & Bali	Precipitation	2.53	0.96	2.41	0.97	-0.01	0.12	TRUE	TRUE	Same
JAWA TENGAH	Java & Bali	Precipitation	1.67	0.95	1.57	0.96	-0.02	0.11	TRUE	TRUE	Same
JAWA TIMUR	Java & Bali	Precipitation	1.18	0.95	1.06	0.98	-0.04	0.13	TRUE	TRUE	Same
KALIMANTAN BARAT	Kalimantan	Precipitation	-0.21	0.82	-0.07	0.88	-0.06	-0.14	FALSE	FALSE	Same
KALIMANTAN SELATAN	Kalimantan	Precipitation	-0.17	0.95	-0.29	0.94	0.01	0.13	FALSE	FALSE	Same
KALIMANTAN TENGAH	Kalimantan	Precipitation	0.02	0.93	-0.03	0.94	-0.01	0.04	TRUE	FALSE	Different
KALIMANTAN TIMUR	Kalimantan	Precipitation	0.75	0.87	0.90	0.90	-0.04	-0.15	TRUE	TRUE	Same
KALIMANTAN UTARA	Kalimantan	Precipitation	5.34	0.32	4.50	0.39	-0.07	0.84	FALSE	FALSE	Same
MALUKU	Maluku	Precipitation	-1.11	0.73	-1.64	0.72	0.01	0.53	FALSE	FALSE	Same
MALUKU UTARA	Maluku	Precipitation	-0.52	0.66	0.10	0.69	-0.03	-0.62	FALSE	FALSE	Same
NUSA TENGGARA BARAT	Nusa Tenggara	Precipitation	1.46	0.95	1.53	0.95	0.00	-0.07	TRUE	TRUE	Same
NUSA TENGGARA TIMUR	Nusa Tenggara	Precipitation	0.41	0.95	0.56	0.96	-0.02	-0.15	TRUE	TRUE	Same
PAPUA	Papua	Precipitation	0.15	0.64	-0.72	0.65	0.00	0.87	FALSE	FALSE	Same
PAPUA BARAT	Papua	Precipitation	-0.20	0.58	-0.08	0.52	0.06	-0.12	FALSE	FALSE	Same
GORONTALO	Sulawesi	Precipitation	-0.67	0.76	-0.31	0.83	-0.08	-0.37	FALSE	FALSE	Same
SULAWESI BARAT	Sulawesi	Precipitation	-0.56	0.59	-0.35	0.74	-0.15	-0.22	FALSE	FALSE	Same
SULAWESI SELATAN	Sulawesi	Precipitation	0.83	0.91	0.60	0.93	-0.02	0.24	TRUE	TRUE	Same
SULAWESI TENGAH	Sulawesi	Precipitation	-2.72	0.59	-2.90	0.52	0.07	0.18	FALSE	FALSE	Same
SULAWESI TENGGARA	Sulawesi	Precipitation	-0.26	0.91	-0.26	0.89	0.02	0.01	FALSE	FALSE	Same
SULAWESI UTARA	Sulawesi	Precipitation	-0.84	0.83	-0.70	0.85	-0.02	-0.14	FALSE	FALSE	Same
ACEH	Sumatra	Precipitation	0.37	0.79	0.32	0.95	-0.16	0.05	FALSE	TRUE	Different
BENGKULU	Sumatra	Precipitation	1.67	0.59	2.14	0.47	0.11	-0.46	FALSE	FALSE	Same
JAMBI	Sumatra	Precipitation	-0.11	0.91	-0.41	0.93	-0.01	0.30	FALSE	FALSE	Same
KEPULAUAN BANGKA BELITUNG	Sumatra	Precipitation	-0.45	0.88	-0.06	0.94	-0.06	-0.39	FALSE	FALSE	Same
KEPULAUAN RIAU	Sumatra	Precipitation	-0.58	0.79	-0.75	0.77	0.01	0.18	FALSE	FALSE	Same
LAMPUNG	Sumatra	Precipitation	0.46	0.90	0.29	0.92	-0.02	0.17	TRUE	TRUE	Same
RIAU	Sumatra	Precipitation	-0.11	0.81	-0.40	0.91	-0.10	0.29	FALSE	FALSE	Same
SUMATRA BARAT	Sumatra	Precipitation	1.76	0.73	1.42	0.88	-0.15	0.34	FALSE	TRUE	Different
SUMATRA SELATAN	Sumatra	Precipitation	0.21	0.96	0.31	0.97	-0.01	-0.10	TRUE	TRUE	Same
SUMATRA UTARA	Sumatra	Precipitation	-0.92	0.87	-0.74	0.93	-0.06	-0.18	FALSE	FALSE	Same
BALI	Java & Bali	Temperature	2.16	0.91	2.17	0.93	-0.02	-0.01	TRUE	TRUE	Same
BANTEN	Java & Bali	Temperature	-5.24	0.37	-4.90	0.88	-0.51	-0.34	FALSE	FALSE	Same
DAERAH ISTIMEWA YOGYAKARTA	Java & Bali	Temperature	0.84	0.82	1.05	0.90	-0.08	-0.20	FALSE	TRUE	Different
DKI JAKARTA	Java & Bali	Temperature	-4.41	0.94	-4.49	0.92	0.01	0.08	FALSE	FALSE	Same
JAWA BARAT	Java & Bali	Temperature	-4.72	0.88	-4.71	0.86	0.02	-0.01	FALSE	FALSE	Same
JAWA TENGAH	Java & Bali	Temperature	-5.98	0.09	5.86	0.17	-0.08	-11.84	FALSE	FALSE	Same
JAWA TIMUR	Java & Bali	Temperature	4.03	0.92	3.79	0.93	-0.01	0.24	TRUE	TRUE	Same
KALIMANTAN BARAT	Kalimantan	Temperature	5.57	0.14	5.87	0.11	0.03	-0.30	FALSE	FALSE	Same
KALIMANTAN SELATAN	Kalimantan	Temperature	4.98	0.40	4.74	0.51	-0.11	0.24	FALSE	FALSE	Same
KALIMANTAN TENGAH	Kalimantan	Temperature	5.43	0.34	5.20	0.45	-0.11	0.22	FALSE	FALSE	Same
KALIMANTAN TIMUR	Kalimantan	Temperature	5.97	0.25	-5.32	0.21	0.04	11.29	FALSE	FALSE	Same
KALIMANTAN UTARA	Kalimantan	Temperature	-2.31	0.54	-2.15	0.86	-0.33	-0.16	FALSE	FALSE	Same
MALUKU	Maluku	Temperature	1.10	0.75	0.47	0.81	-0.05	0.63	FALSE	FALSE	Same
MALUKU UTARA	Maluku	Temperature	1.70	0.51	1.54	0.45	0.06	0.17	FALSE	FALSE	Same
NUSA TENGGARA BARAT	Nusa Tenggara	Temperature	2.69	0.91	2.67	0.91	0.00	0.02	TRUE	TRUE	Same
NUSA TENGGARA TIMUR	Nusa Tenggara	Temperature	1.97	0.93	1.95	0.95	-0.02	0.02	TRUE	TRUE	Same
PAPUA	Papua	Temperature	1.58	0.67	0.80	0.65	0.02	0.78	FALSE	FALSE	Same
PAPUA BARAT	Papua	Temperature	1.76	0.56	1.28	0.53	0.04	0.48	FALSE	FALSE	Same
GORONTALO	Sulawesi	Temperature	3.92	0.60	4.18	0.66	-0.06	-0.26	FALSE	FALSE	Same
SULAWESI BARAT	Sulawesi	Temperature	0.25	0.54	0.58	0.65	-0.10	-0.32	FALSE	FALSE	Same
SULAWESI SELATAN	Sulawesi	Temperature	3.57	0.90	3.40	0.89	0.01	0.16	TRUE	TRUE	Same
SULAWESI TENGAH	Sulawesi	Temperature	1.55	0.69	0.84	0.71	-0.02	0.71	FALSE	FALSE	Same
SULAWESI TENGGARA	Sulawesi	Temperature	2.45	0.85	2.46	0.82	0.04	-0.01	TRUE	FALSE	Different
SULAWESI UTARA	Sulawesi	Temperature	1.52	0.70	0.96	0.72	-0.03	0.56	FALSE	FALSE	Same
ACEH	Sumatra	Temperature	5.78	0.20	5.82	0.17	0.03	-0.04	FALSE	FALSE	Same
BENGKULU	Sumatra	Temperature	-1.89	0.62	-2.84	0.62	0.00	0.95	FALSE	FALSE	Same
JAMBI	Sumatra	Temperature	5.87	0.09	5.51	0.23	-0.14	0.36	FALSE	FALSE	Same
KEPULAUAN BANGKA BELITUNG	Sumatra	Temperature	-5.96	0.30	-5.65	0.28	0.01	-0.31	FALSE	FALSE	Same
KEPULAUAN RIAU	Sumatra	Temperature	4.01	0.50	4.43	0.39	0.11	-0.42	FALSE	FALSE	Same
LAMPUNG	Sumatra	Temperature	5.35	0.49	5.39	0.52	-0.03	-0.04	FALSE	FALSE	Same
RIAU	Sumatra	Temperature	5.77	0.16	5.45	0.26	-0.11	0.31	FALSE	FALSE	Same
SUMATRA BARAT	Sumatra	Temperature	-4.01	0.50	-4.27	0.87	-0.38	0.27	FALSE	FALSE	Same
SUMATRA SELATAN	Sumatra	Temperature	5.36	0.41	5.52	0.30	0.11	-0.16	FALSE	FALSE	Same
SUMATRA UTARA	Sumatra	Temperature	4.27	0.88	4.52	0.92	-0.04	-0.25	TRUE	TRUE	Same

[1]F = Full (2010-2024); PP = Pre-pandemic (2010-2019); Coh = Coherence; Qual = Qualified for DLNM

2.2.3 Table J: DLNM Cumulative Relative Risk Comparison

Comparison of DLNM-derived cumulative relative risks at wavelet-derived lags between full dataset and pre-pandemic period. Only provinces qualifying in the full period are shown.

Table 10

Table J. DLNM Relative Risk Sensitivity to COVID-19 Period[1]

Prov.	Clim.	L(F)	RR(F)	RRL(F)	RRH(F)	Sig(F)	L(PP)	RR(PP)	RRL(PP)	RRH(PP)	Sig(PP)	ΔRR	Both Sig	Dir Agree
BALI	Precipitation	3	1.18	0.72	1.94	No	2	1.26	0.71	2.23	No	-0.07	TRUE	Same direction
BANTEN	Precipitation	2	1.13	0.77	1.66	No	2	1.92	1.40	2.64	Yes	-0.79	FALSE	Same direction
DAERAH ISTIMEWA YOGYAKARTA	Precipitation	2	1.59	1.01	2.48	Yes	2	0.67	0.45	0.99	Yes	0.91	TRUE	Different direction
DKI JAKARTA	Precipitation	3	1.59	1.04	2.43	Yes	2	2.65	1.81	3.89	Yes	-1.07	TRUE	Same direction
JAMBI	Precipitation	0	1.28	1.01	1.62	Yes	0	1.12	0.86	1.46	No	0.16	FALSE	Same direction
JAWA BARAT	Precipitation	3	1.15	0.78	1.70	No	2	0.84	0.61	1.14	No	0.32	TRUE	Different direction
JAWA TENGAH	Precipitation	2	1.33	0.88	2.02	No	2	0.65	0.40	1.04	No	0.69	TRUE	Different direction
JAWA TIMUR	Precipitation	1	0.94	0.68	1.29	No	1	0.62	0.45	0.84	Yes	0.32	FALSE	Same direction
KALIMANTAN SELATAN	Precipitation	0	1.48	1.14	1.92	Yes	0	1.35	1.04	1.76	Yes	0.13	TRUE	Same direction
KALIMANTAN TENGAH	Precipitation	0	1.65	1.37	1.99	Yes	0	1.15	0.94	1.40	No	0.51	FALSE	Same direction
KALIMANTAN TIMUR	Precipitation	1	1.28	1.09	1.51	Yes	1	1.22	0.96	1.55	No	0.06	FALSE	Same direction
KEPULAUAN BANGKA BELITUNG	Precipitation	0	1.46	1.21	1.76	Yes	0	1.46	1.17	1.82	Yes	0.00	TRUE	Same direction
LAMPUNG	Precipitation	0	1.30	1.06	1.59	Yes	0	1.08	0.89	1.31	No	0.22	FALSE	Same direction
NUSA TENGGARA BARAT	Precipitation	1	1.95	1.38	2.75	Yes	2	2.30	1.09	4.84	Yes	-0.35	TRUE	Same direction
NUSA TENGGARA TIMUR	Precipitation	0	1.60	0.95	2.69	No	1	5.58	1.93	16.12	Yes	-3.98	FALSE	Same direction
SULAWESI SELATAN	Precipitation	1	1.33	1.05	1.67	Yes	1	1.10	0.84	1.43	No	0.23	FALSE	Same direction
SULAWESI TENGGARA	Precipitation	0	1.12	0.94	1.35	No	0	1.20	0.98	1.47	No	-0.08	TRUE	Same direction
SUMATRA SELATAN	Precipitation	0	1.21	1.06	1.38	Yes	0	1.14	0.98	1.33	No	0.06	FALSE	Same direction
BALI	Temperature	2	0.85	0.62	1.18	No	2	1.37	0.68	2.75	No	-0.51	TRUE	Different direction
JAWA TIMUR	Temperature	4	1.24	0.83	1.85	No	4	11.79	5.90	23.56	Yes	-10.55	FALSE	Same direction
NUSA TENGGARA BARAT	Temperature	3	0.60	0.39	0.93	Yes	3	1.48	0.71	3.06	No	-0.88	FALSE	Different direction
NUSA TENGGARA TIMUR	Temperature	2	1.49	0.85	2.60	No	2	2.13	0.99	4.55	No	-0.64	TRUE	Same direction
SULAWESI SELATAN	Temperature	4	3.36	1.71	6.61	Yes	3	0.81	0.39	1.68	No	2.55	FALSE	Different direction
SULAWESI TENGGARA	Temperature	2	1.12	0.75	1.66	No	2	0.48	0.26	0.90	Yes	0.63	FALSE	Different direction

[1]F = Full (2010-2024); PP = Pre-pandemic (2010-2019); L = Lag; RRL/RRH = RR Lower/Higher CI; Sig = Significant

2.3 Interpretation

The sensitivity analysis shows that our key findings are **robust to the inclusion of pandemic years**. Phase lag directions remain consistent for the majority of provinces, and provinces qualifying for DLNM analysis show substantial overlap between periods. While absolute values of phase coherence and relative risk estimates differ slightly—expected given the shorter time series in the pre-pandemic period—the qualitative conclusions regarding which provinces show reliable climate-dengue timing relationships and elevated dengue risk remain unchanged.

This robustness suggests that the temporal patterns we identify reflect genuine epidemiological dynamics rather than surveillance artifacts, though we acknowledge that case detection likely declined during 2020-2021 and subsequent recovery may have introduced noise.

Table 11

Table K. Sensitivity of Results to Phase Coherence Threshold[1]

threshold	clim_var	n	n_significant	pct_significant
0.70	Precipitation	22	12	54.5
0.70	Temperature	9	3	33.3
0.75	Precipitation	21	11	52.4
0.75	Temperature	9	3	33.3
0.80	Precipitation	20	11	55.0
0.80	Temperature	8	3	37.5
0.85	Precipitation	18	11	61.1
0.85	Temperature	7	3	42.9
0.90	Precipitation	16	9	56.2
0.90	Temperature	5	2	40.0

[1]Bold row indicates threshold used in main analysis (0.85)

3 Phase Coherence Threshold Sensitivity Analysis

Our main analysis uses a phase coherence threshold of 0.85 to identify provinces where climate variables show reliable timing relationships with dengue. This threshold represents a high degree of consistency in the climate-dengue phase relationship. To assess whether our conclusions are sensitive to this threshold choice, we tested alternative thresholds ranging from 0.70 to 0.90.

3.1 Methods

For each threshold value (0.70, 0.75, 0.80, 0.85, 0.90), we:

1. Identified provinces meeting the threshold criteria (coherence threshold AND phase lag 0)
2. Counted qualifying provinces for precipitation and temperature
3. Determined how many qualifying provinces showed statistically significant DLNM effects (cumulative RR confidence interval excluding 1.0)

Higher thresholds are more stringent, selecting only provinces with very consistent climate-dengue timing. Lower thresholds are more permissive but may include provinces where the relationship is less reliable.

3.2 Results

3.2.1 Table K: Threshold Sensitivity Analysis

3.3 Interpretation

The number of qualifying provinces decreases predictably as the threshold becomes more stringent. The table shows that stricter coherence requirements substantially reduce the number of qualifying provinces, but the number showing statistically significant DLNM effects remains relatively stable, particularly for the 0.75-0.85 range.

Importantly, **the number of provinces showing statistically significant DLNM effects remains relatively stable** across thresholds. This indicates that our choice of 0.85 is conservative but does not substantially affect the core finding: provinces with reliable climate-dengue timing also tend to show significant dose-response relationships in DLNM models.

The 0.85 threshold balances two considerations: (1) not excluding too many provinces for meaningful interpretation, and (2) maintaining high confidence in the reliability of the climate-dengue timing relationship.

4 Supplementary Figures

4.1 Figure A: Geographical Regions of Indonesia

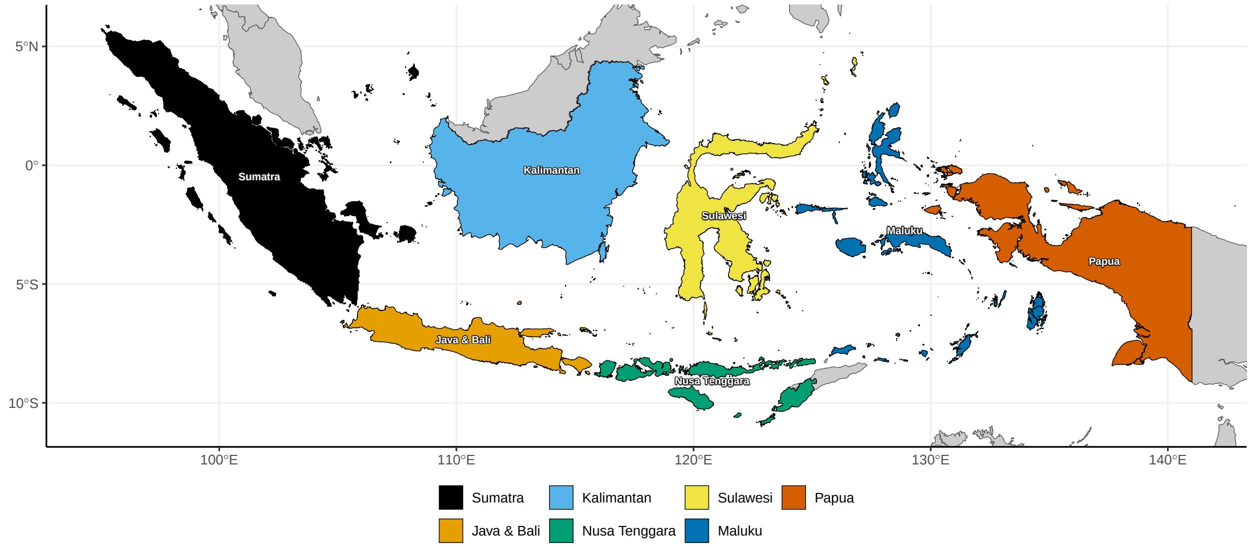

Figure 1: Geographical regions of Indonesia used for regional stratification in this study. Regions follow standard Indonesian geographic divisions. Administrative boundaries from geoBoundaries (Runfola et al. 2020 [38], doi:10.1371/journal.pone.0231866), CC BY 4.0.

4.2 Figure B: Province Phase Lags with Confidence Intervals

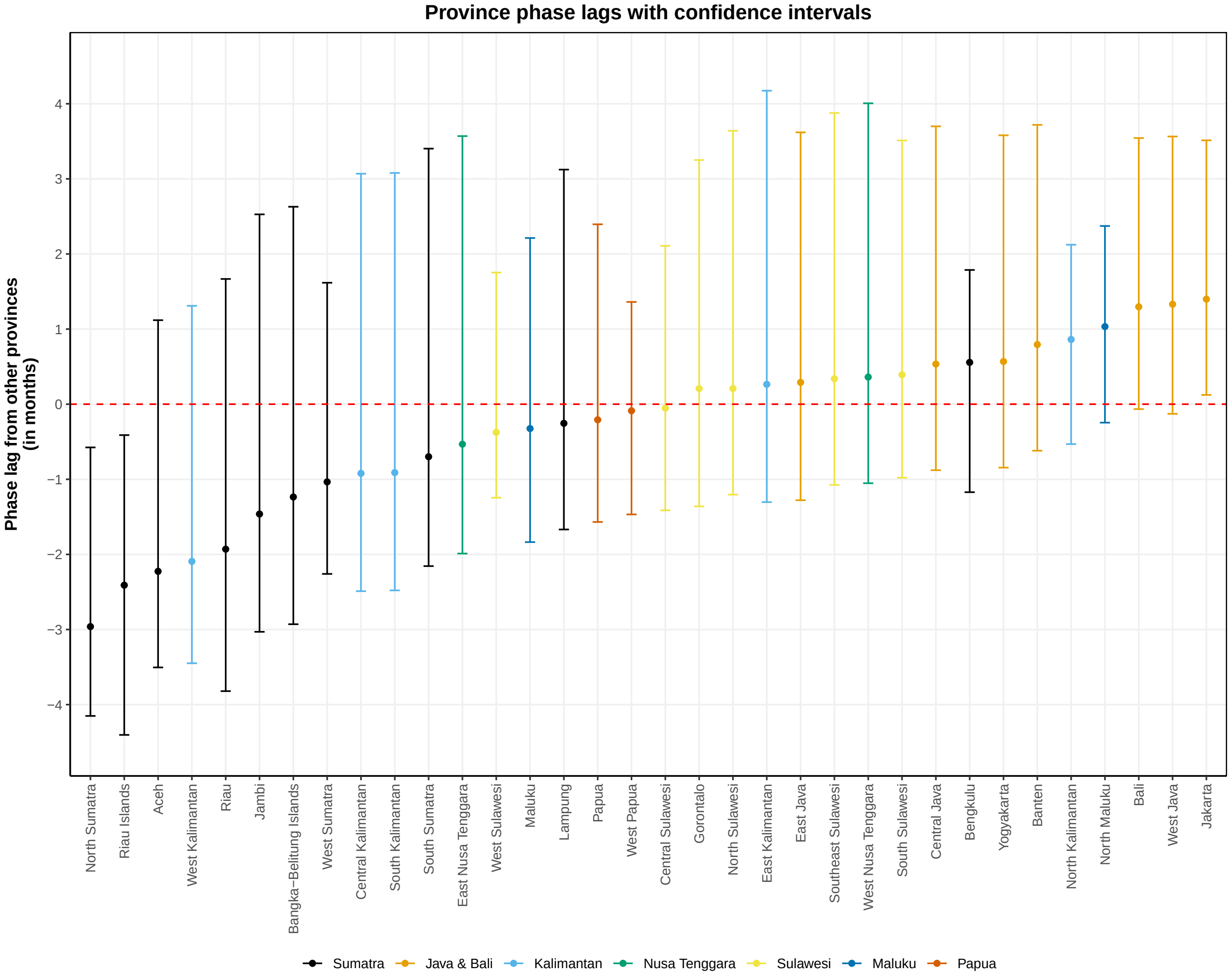

Figure 2: Between-province phase lags with 2.5th-97.5th percentile ranges of pairwise differences. Positive values indicate provinces with later peaks relative to other provinces; negative values indicate earlier peaks. Error bars represent the range of phase differences with all other provinces, not statistical uncertainty.

4.3 Figure C: Comparison of Wavelet-Based and DLNM-Based Lag Estimates

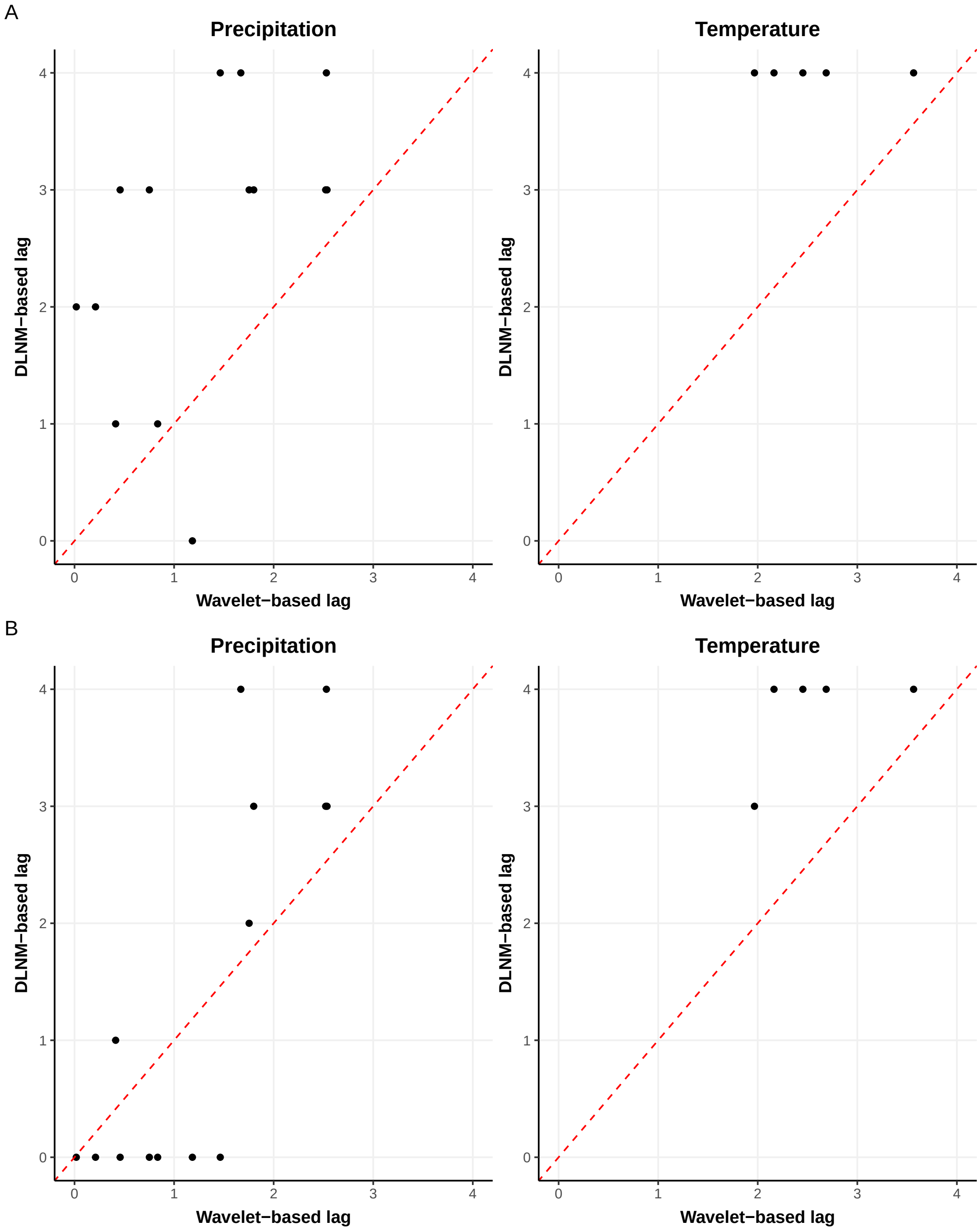

Figure 3: Comparison between wavelet-derived phase lags and DLNM-based optimal lags for precipitation and temperature. Panel A shows DLNM lags selected using the 'highest-risk' approach (lag with maximum cumulative RR); Panel B shows DLNM lags selected using the 'first-significant' approach (earliest lag with significant elevated risk). The red dashed line indicates perfect agreement. Divergence between methods is expected because wavelet phase assumes sinusoidal cycles while DLNM allows non-linear dose-response relationships.

4.4 Figure D: Year-by-Year Peak Month Variability (All Provinces)

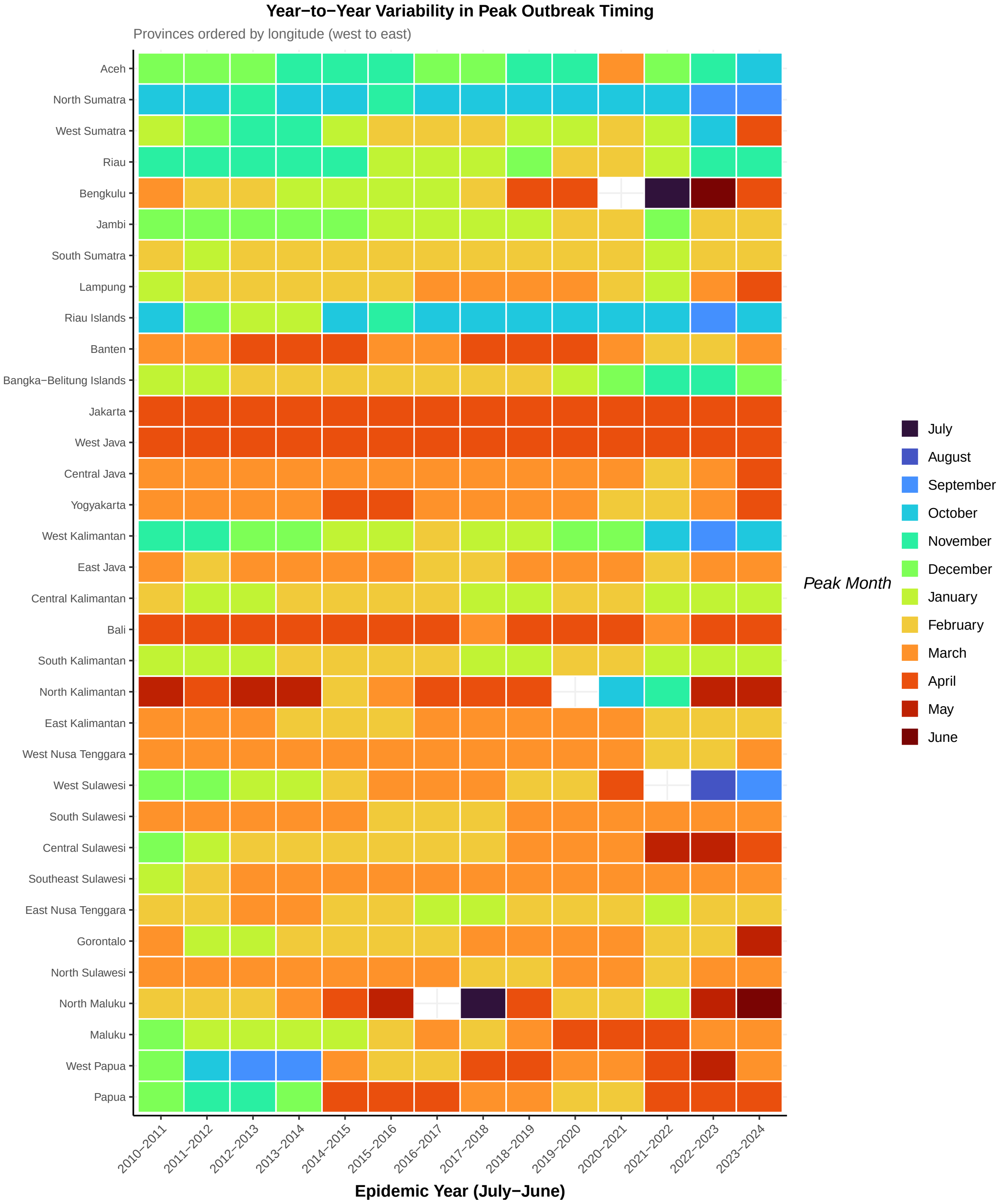

Figure 4: Year-to-year variability in peak outbreak timing across all 34 provinces. Provinces are ordered by longitude (west to east, bottom to top). Each cell shows the peak month for that province in that epidemic year (July-June). Grey cells indicate anomalous periods excluded due to weak annual periodicity. The heatmap reveals substantial inter-annual variability in peak timing, even within the same province, highlighting the complexity of dengue seasonality across Indonesia's archipelagic geography.

4.5 Figure E: Year-by-Year Peak Month Variability (Western Provinces)

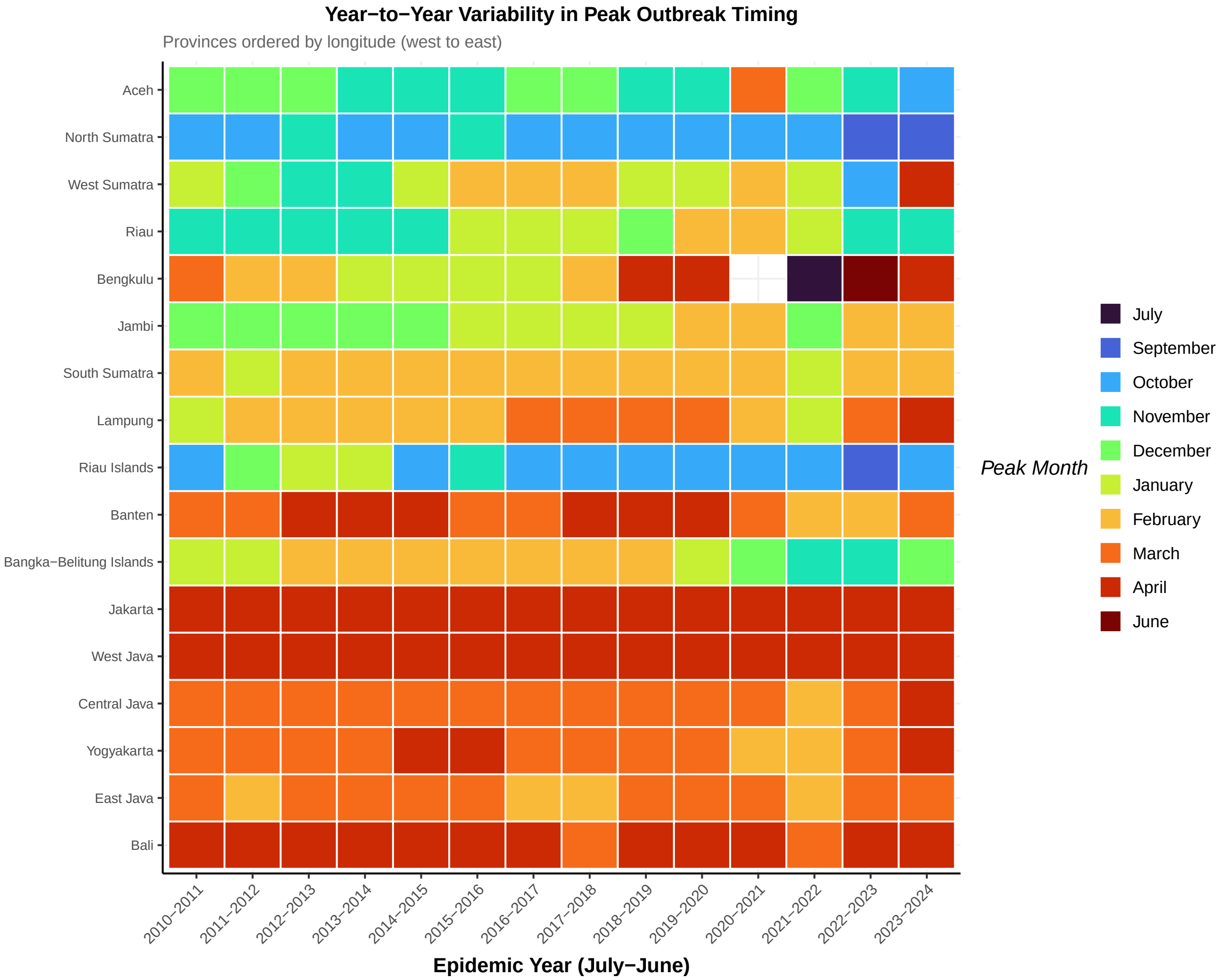

Figure 5: Year-to-year variability in peak outbreak timing for western provinces only (Sumatra and Java-Bali). Provinces are ordered by longitude (west to east, bottom to top). This subset shows the regions with clearest cluster structure (Figure 3C in main text). Despite stronger regional coherence than eastern provinces, substantial year-to-year variation in peak timing persists.
