# Supplementary material for: Dengue transmission heterogeneity across Indonesia’s archipelago: Climate-driven spatiotemporal patterns and policy implications
Source: PLoS Negl Trop Dis. 2026 Mar 17;20(3):e0014135. doi: 10.1371/journal.pntd.0014135 (PMC13004528; doi:10.1371/journal.pntd.0014135)
Supplement: Attachment — Submitted filename: Dengue-climate Indonesia_response-to-reviewers.docx [file pntd.0014135.s003.docx]

## **Response to Reviewers**

**Manuscript ID:** PNTD-D-25-01679

**Title:** Dengue transmission heterogeneity across Indonesia’s archipelago: climate-driven spatiotemporal patterns and policy implications

We thank the Editor and Reviewers for their thorough and constructive feedback. These comments have substantially strengthened our manuscript. In response, we have:

1. Extended the study period from 2016–2024 to 2010–2024 (15 years)
2. Added distributed lag non-linear models (DLNM) for exposure-response quantification
3. Added Indian Ocean Dipole (DMI) analysis with regional breakdown
4. Added dynamic time warping (DTW) clustering for temporal similarity assessment
5. Conducted COVID-19 sensitivity analyses and phase coherence threshold sensitivity analyses
6. Removed the surveillance capacity adjustment and urbanisation analyses (rationale provided below)
7. Restructured the Discussion around three principal findings with shortened, focused interpretation

Below we provide point-by-point responses. Reviewer comments are in **bold**, and our responses follow in regular text.

### **Reviewer 1**

We thank Reviewer 1 for their comprehensive evaluation. Many comments relate to methodological rigour in causal inference. We address these in thematic groups, noting that our revised framing explicitly positions this as an exploratory characterisation study rather than a causal analysis.

**1.1. The manuscript does not provide explicit exposure–response functions linking precipitation, temperature, and relative humidity to dengue incidence, nor does it quantify lagged, non-linear effects with effect sizes and uncertainty.**

We have added distributed lag non-linear models (DLNM, as per Reviewer 2 suggestion) to quantify exposure-response relationships. For provinces with reliable climate-dengue timing (phase coherence ≥0.85 and positive lag), we estimated cumulative relative risks comparing the 90th to 50th percentile of precipitation and temperature at wavelet-derived optimal lags. Results show that 11 of 18 qualifying provinces had significantly elevated risk with high precipitation (cumulative RR increases of 21–95%), while 3 of 7 qualifying provinces showed significant temperature effects. We now report effect sizes with 95% confidence intervals and discuss how DLNM complements wavelet analysis by quantifying magnitude rather than just timing. [Methods, "Distributed lag non-linear models"; Results, paragraph 6; Discussion, "Complementarity of wavelet and DLNM approaches"; Fig 6]

**1.2–1.3. Confounding structures (trends, seasonality, ENSO, surveillance changes, COVID-19) were not adequately handled. The manuscript does not demonstrate that observed patterns are not artefacts of detection variance.**

We acknowledge these important concerns and have made several changes:

*Analytical adjustments:* We added DLNM as one of our analytical tools in this study. We focused on provinces with high phase coherence (≥0.85), which inherently selects for provinces with consistent climate-dengue relationships less likely to be dominated by surveillance noise.

*COVID-19 sensitivity:* We conducted sensitivity analyses excluding 2020–2021, showing that phase lag estimates, phase coherence values, and DLNM associations were qualitatively similar to full-period results (Tables S8–S10).

*Surveillance heterogeneity:* Rather than attempting inadequately validated adjustments, we now explicitly acknowledge surveillance heterogeneity as a limitation and note that the west-to-east gradient was strongest in regions with higher estimated surveillance capacity (Sumatra and Java-Bali), suggesting the pattern may reflect genuine epidemiological dynamics. We have removed the surveillance capacity adjustment analysis (see response to Comments 1.3 and 2.2 below for rationale).

*Reframed interpretation:* The revised manuscript explicitly positions this as exploratory characterisation, with the opening Discussion sentence now reading: "This exploratory study characterised spatiotemporal heterogeneity in dengue incidence across Indonesia's 34 provinces over 15 years (2010-2024) and examined relationships with climate variables."

[Methods throughout; Limitations, paragraphs 2–4; Tables S8–S10]

**1.4. The manuscript does not provide documentation of case definition shifts nor how they were addressed.**

Indonesia's dengue surveillance has used consistent clinical case definitions for dengue haemorrhagic fever (DHF) throughout the study period, based on WHO 1997 criteria. We now state this explicitly and acknowledge that while case definitions have been consistent, laboratory confirmation capacity and reporting completeness have likely improved over time, particularly in western provinces. [Limitations, paragraph 2]

**1.5. The provenance, resolution, preprocessing, and provincial aggregation of climate data are not sufficiently detailed. The manuscript does not assess sensitivity to alternative climate products.**

We have expanded the Methods to detail climate data processing: ERA5-Land data were extracted and aggregated to provincial level using population-weighted averaging based on LandScan Global Population Database. We acknowledge that we did not conduct sensitivity analysis to alternative climate products (e.g., CHIRPS for precipitation), which represents a limitation. However, ERA5-Land is widely used for climate-health studies and provides consistent coverage across our study period. [Methods, "Climate data"]

**1.6. The document does not clearly disentangle basin-scale ENSO effects from local climate associations.**

We now explicitly distinguish these scales analytically and interpretively. We focused on local climate (precipitation, temperature) for phase and dose-response analyses because they operate at the annual timescale matching the 12-month dengue cycles extracted by wavelet analysis. ENSO/IOD indices capture multi-annual cycles and are analysed separately for strategic (rather than tactical) early warning. We acknowledge in the limitations that we did not model ENSO within the DLNM framework, though such indices may improve predictive power in multivariate models. [Discussion, "Climate-dengue relationships at two timescales"; Limitations, paragraph 4]

**1.7. Statistical inference in the presence of strong autocorrelation is not adequately justified.**

We now explicitly acknowledge this limitation. The Spearman correlation between ONI and epidemic-year incidence is based on a small sample size (n = 14 epidemic years), and temporal autocorrelation in monthly time series may reduce effective sample sizes and inflate apparent significance. We state that p-values in Table 1 should be interpreted as nominal values given potential autocorrelation structure. [Limitations, paragraph 4; Table 1 caption]

**1.8. The phase and coherence analyses lack detail on key methodological choices and uncertainty quantification.**

We have expanded the Methods on wavelet analysis. Phase lags are reported with 2.5th–97.5th percentile ranges from pairwise comparisons (Fig S2). The phase coherence threshold of 0.85 is acknowledged as arbitrary, with sensitivity analysis across 0.70–0.90 thresholds showing stable results (Table S11). [Methods, "Wavelet analysis"; Fig S2; Table S11; Limitations, paragraph 5]

**1.9. The definition of incidence denominators and handling of population dynamics are not fully transparent.**

Provincial populations for intercensal years were estimated using linear interpolation and extrapolation by age group and sex between the 2010 and 2020 censuses [Methods, "Epidemiological data"]

**1.10. The manuscript reports relationships involving urban density but lacks mechanistic justification and evaluation of effect modification.**

We have removed the urbanisation analysis from the revised manuscript. The original analysis had fundamental metric issues (the "urban density" definition was not dimensionally intuitive and potentially collinear with surveillance capacity), and addressing these properly would require a multivariable spatial regression framework with additional covariates (healthcare access, socioeconomic indicators) that was beyond the scope of this climate-focused exploratory analysis. We believe urbanisation–dengue relationships in Indonesia warrant dedicated investigation with appropriate data and methods. The revised manuscript now focuses on characterising climate–dengue timing relationships, which it can do well with the available data.

**1.11. The manuscript does not undertake formal attribution analysis for human mobility versus climate variability.**

We agree this is an important gap. We have added dynamic time warping (DTW) clustering to distinguish temporal similarity patterns, and we now use correlation tests to see whether the phase gradient is formally associated with longitude. However, we lack mobility data (e.g., mobile phone records, transport networks) to directly quantify mobility contributions. We now frame the west-to-east gradient interpretation more cautiously, noting it "suggests that provincial differences in peak timing reflect climate-driven seasonality rather than sequential disease spread between provinces" [Discussion, "West-to-east gradient"; Limitations]

### **Reviewer 2**

**2.1. The current data availability statement may not meet PLOS's requirement. Province-by-month aggregated incidence time series should be openly shared.**

We have revised the data availability statement. Province-by-month aggregated dengue incidence data for 2010–2024 will be deposited in <https://github.com/mlgh-sg/dengue-climate-indonesia> upon acceptance. They are also available from OpenDengue. Raw surveillance records remain subject to Ministry of Health data sharing policies. [Data Availability Statement]

**2.2. The surveillance "capacity" index adjustment procedure is not specified. Include surveillance capacity as a covariate in a multivariable framework.**

We have removed the surveillance capacity adjustment from the revised manuscript. The original adjustment (dividing reported incidence by the capacity index) was methodologically problematic: (1) the Lim et al. estimates were derived from a global model without subnational validation in Indonesia, (2) dividing by values near zero produces unstable estimates, and (3) a simple scalar adjustment does not propagate uncertainty. Properly addressing surveillance heterogeneity would require either advanced modelling of true incidence and reporting probability, or validated subnational surveillance indicators, neither of which was available.

Instead, we now acknowledge surveillance heterogeneity as an explicit limitation and note that our findings were strongest in higher-capacity regions (Sumatra, Java-Bali), suggesting the climate-dengue patterns reflect genuine epidemiological dynamics. Future work incorporating validated surveillance covariates in a multivariable framework is warranted but beyond the current scope. [Limitations, paragraph 2]

**2.3. Using ONI alone risks missing the role of the Indian Ocean Dipole (IOD). Add DMI and use DLNM to quantify exposure-response curves.**

We have addressed both points:

*IOD/DMI:* We now include DMI analysis alongside ONI. At the national level, DMI showed weaker association with epidemic-year incidence ($\rho$ = 0.33, p = 0.25) than ONI ($\rho$ = 0.83, p < 0.01). However, regional patterns differed: DMI showed stronger associations than ONI in Maluku ($\rho$ = 0.60 vs 0.29) and Nusa Tenggara ($\rho$ = 0.46 vs 0.32) (Table S7). We discuss how this regional heterogeneity may partially explain the breakdown of the west-to-east gradient in eastern Indonesia.

*DLNM:* We have added DLNM analysis for local climate variables (precipitation, temperature), reporting cumulative relative risks with 95% CIs for provinces with high phase coherence. We discuss how wavelet phase analysis and DLNM provide complementary information: phase coherence screens for reliable timing relationships, while DLNM quantifies effect magnitude.

We did not model ONI/DMI within the DLNM framework because these indices operate at multi-annual timescales distinct from the annual climate-dengue cycles. We acknowledge this as a limitation and note that such indices may improve predictive power in multivariate models.

[Results, paragraph 4; Discussion, "Climate-dengue relationships at two timescales" and "West-to-east gradient"; Table S7; Limitations, paragraph 4]

**2.4. "Urban density" definition is problematic and may be collinear with surveillance capacity. Use standard population density and include in multivariable spatial regression.**

We have removed the urbanisation analysis entirely. The reviewer correctly identifies that our metric (urban population share / urban land share) was not dimensionally intuitive, sensitive to small denominators, and potentially collinear with surveillance capacity and development indicators. Addressing this properly would require: (1) standard density metrics from GHS-POP or WorldPop, (2) multivariable spatial regression with covariates for surveillance, healthcare access, WASH, and socioeconomic status, and (3) stratified analyses (Java vs. non-Java). This would substantially change the scope toward a spatial epidemiology paper rather than the climate-exploratory focus we intended.

We believe urbanisation–dengue relationships in Indonesia are important but warrant dedicated investigation with appropriate methods. The revised manuscript focuses on exploring climate–dengue relationships. [See response to 1.10]

**2.5. Population interpolation ignores age structure and migration; COVID-19 altered care-seeking and surveillance.**

We acknowledge these limitations. For population, we now state explicitly that interpolation accounts for age structure and sex. For COVID-19, we conducted sensitivity analyses excluding 2020–2021, showing qualitatively similar results for phase lags, coherence, and DLNM associations (Tables S8–S10). We did not include mobility or policy stringency covariates as these would require a different analytical framework focused on pandemic effects rather than climate relationships. [Methods; Limitations, paragraph 3; Tables S8–S10]

**2.6. Test the phase gradient formally via circular-linear regression and use DTW to distinguish climate forcing from inter-provincial propagation.**

We have addressed both points:

*Formal phase gradient testing:* We tested the association between peak month and longitude using Spearman rank correlation in each epidemic year (July-June period), confirming significant west-to-east gradients, especially when only Western provinces were included. [Results, paragraph 2; Methods]

*DTW clustering:* We added dynamic time warping analysis to assess temporal similarity. Results show that all 34 provinces require 12 clusters for adequate separation, while restricting to western provinces improves coherence. This supports our interpretation that geographic proximity does not guarantee similar outbreak dynamics, and that the gradient reflects differential climate timing rather than sequential spread—provinces cluster by climate regime rather than geographic contiguity.

[Methods, "Dynamic time warping"; Results, paragraph 3; Discussion, "Heterogeneity within geographical regions"; Tables S3–S6]

**2.7. Given anomalously low 2020 incidence, include intervention/indicator variables in main models.**

We conducted sensitivity analyses excluding 2020 onwards rather than including intervention covariates. This approach was chosen because: (1) Google Mobility and policy stringency indices would require a different analytical framework focused on pandemic effects, (2) our focus is climate-dengue relationships rather than pandemic impacts, and (3) exclusion sensitivity analysis directly tests whether pandemic years drive our conclusions (they do not—results were qualitatively similar). [Limitations, paragraph 3; Tables S8–S10]

**2.8. Add a reproducible worked example linking climate-phase-intervention for two contrasting regions with quantified case reductions.**

We have added operational guidance in the Discussion, using North Sumatra (early-peaking, October) and Central Java (later-peaking, February) as illustrative examples. We describe how precipitation signals at province-specific lead times (1–3 months based on DLNM optimal lags) could inform intervention timing. However, we stopped short of quantifying case reductions via counterfactual simulation, as this would require: (1) validated transmission models for each province, (2) intervention effectiveness estimates, and (3) prospective validation data—none of which are available. We emphasise that prospective operational validation is essential before implementation.

We believe providing a hypothetical case reduction estimate without such validation could be misleading and prefer to clearly state what prospective research is needed. [Discussion, "Implications for early warning"]

### **Reviewer 3**

**3.1. The surveillance capacity adjustment lacks validation at subnational level in Indonesia. Include comparison of adjusted vs unadjusted incidence and sensitivity analysis using unadjusted rates.**

We have removed the surveillance capacity adjustment entirely from the revised manuscript. We agree with the reviewer that the Lim et al. global estimates lack subnational validation in Indonesia, and that the adjustment dramatically altered province-level incidence values without adequate justification.

Rather than attempting to fix an inadequately validated adjustment, we now: (1) present unadjusted incidence throughout, (2) explicitly acknowledge surveillance heterogeneity as a limitation, (3) note that eastern provinces likely experience greater underreporting, and (4) observe that the west-to-east gradient was strongest in higher-capacity regions (Sumatra, Java-Bali), suggesting the pattern may reflect genuine epidemiological dynamics rather than surveillance artifacts.

This approach is more transparent about data limitations while avoiding spurious precision from unvalidated adjustments. [Limitations, paragraph 2]

**3.2. The calculation of 'urban population density' is unclear and the association with dengue incidence is oversimplified and misrepresented.**

We have removed the urbanisation analysis entirely. The reviewer correctly identifies multiple issues: (1) our metric did not represent population density as typically understood, (2) the surveillance adjustment that preceded correlation analysis was not validated, and (3) reporting a linear correlation for highly skewed data was inappropriate. See our response to Reviewer 2, Comment 2.4 for the full rationale. The revised manuscript no longer includes urbanisation analyses or claims. [See response to 2.4]

**3.3. The phase lag analysis has huge variability across provinces and/or years. Report year-to-year variability explicitly to understand whether the west-to-east progression is consistent.**

We have added Figures S4 and S5 showing year-by-year peak month for all 34 provinces and western provinces only, respectively. These figures reveal substantial interannual variability in peak timing, as the reviewer anticipated. However, the west-to-east gradient remains visible in most years for western provinces, even if individual province rankings shift.

We now reference these figures in the Results and acknowledge that the phase lag represents a central tendency with considerable year-to-year variation. The error bars in Figure S2 (moved from the main text to the Appendix) show 2.5th–97.5th percentile ranges from pairwise phase comparisons, which reflect both interprovincial and interannual variability. [Results, paragraph 2; Figures S4–S5]

**3.4. The coefficient of variation reflects interannual variability as well as seasonality.**

The reviewer is correct that CoV calculated from monthly time series conflates within-year seasonality and between-year amplitude variation, limiting its interpretability. We have removed the bivariate maps from the revised manuscript. The revised analysis focuses on phase relationships and dose-response associations, which more directly address the climate-dengue timing relationships central to our research questions.

**3.5. How does the recommendation for province-specific timing correspond to Indonesia's current strategy? Are interventions already decentralised?**

We have revised this section to acknowledge Indonesia's existing decentralised structure. Indonesia's dengue control is largely decentralised, with provincial and district health offices implementing interventions based on local trends, while the national government provides coordination through the 2021–2025 National Strategic Plan for Dengue Control. Our findings support this structure but suggest that inter-provincial coordination could optimise resource deployment—earlier-peaking provinces could inform preparedness in later-peaking neighbours. [Discussion, "Implications for early warning"]

**3.6. The discussion should be considerably shortened with less overinterpretation. The statement about climate-driven timing versus disease spread contradicts later statement about mobility.**

We have substantially revised and shortened the Discussion, reducing speculative statements. For the specific contradiction noted: we now frame this more carefully. The west-to-east gradient "suggests that provincial differences in peak timing reflect climate-driven seasonality rather than sequential disease spread between provinces". [Discussion throughout]

**3.7. For all maps, move labels off provinces in Java for visibility.**

We have revised all maps to change province labels with index, which is referenced in Table S1. [Figures 1–5]

**3.8. Figure 1C uses different scaling than Figure 1A but same colour range. Clarify normalisation method.**

We have revised Figure 1 to include a separate colour legend for panel C, clearly indicating that it shows z-score normalised monthly incidence (zero mean, unit variance across all province-months, not within provinces). The Methods now explicitly state the normalisation procedure. [Figure 1; Methods]

### **Minor Comments (Reviewer 3)**

We thank the reviewer for the minor comments and have addressed them accordingly.
